# 

**Published:** 1895-04

**Authors:** 


### CONTENTS:

### SELECTIONS.

Article I.—The Immediate Treatment of the Uncovered Pulp. By Prof. Louis Jack, - - - - - - 529
" II.—Tin and Its Combinations with Gold and Amalgam. By S. B. Palmer, M. D. S., - - - - 538
" III.—Vulcanizing Rubber. By Dr. Geo. B. Snow, - - 545
" IV.—Mineral Cements. By Prof. S. J. Willey, - - 547
" V.—Fractures of the Maxilla. By Dr. P. L. Haight, - 551
" VI.—Porcelain Dental Art. W. A. Capon, D. D. S., - 554
" VII.—Sulfuric Acid for Opening Root-Canals, - - 564

### EDITORIAL, ETC.

The Census Bulletin, - - - - - - - - 567
The New Gas "Argon" and its Discovery, - - - - 568

### MONTHLY SUMMARY.

Utilizing Amalgam Waste, - - - - - - - - 571

Tongue in Disease, - - - - - - - - 573

Delusive Pain, - - - - - - - - 574

Magnesium Hydrate, - - - - - - - 574

The Advent of Interruptions, - - - - - - - 575

What Part Did Antitoxin Play? - - - - - - 575

Hydrogen Dioxide as a Blood-Test, - - - - - 576

Bleachant, - - - - - - - - - 576

To Stop a Hemorrhage from Tooth Extraction, - - - 576

